# Effect of soft Chinese martial arts on depressive symptoms among college students: a systematic review and meta-analysis

**DOI:** 10.3389/fpsyt.2026.1850402

**Published:** 2026-10-20

**Authors:** Longlong Shi, Qianjing Wang, Wenyi Li, Wei Xie

**Affiliations:** 1 School of Dance and Martial Arts, Capital University of Physical Education and Sports, Beijing, China; 2 Sports Institute, North Minzu University, Yinchuan, China; 3 Department of Sports, University of Electronic Science and Technology of China, Chengdu, China; 4 School of Physical Education, Guangdong University of Technology, Guangzhou, China

**Keywords:** college students, depression, martial arts, meta-analysis, systematic review

## Abstract

**Objectives:**

Mental health problems are a growing concern among college students worldwide. While exercise is a proven mental health intervention, a quantitative synthesis on the effects of soft Chinese martial arts, as a unique mind-body practice, on depression in this specific population is lacking. This study aimed to fill this gap by systematically evaluating the effectiveness of soft Chinese martial arts interventions in alleviating depressive symptoms among college students.

**Methods:**

Literature with publication dates up to July 31, 2025 was searched in seven databases, including PubMed/MEDLINE, Embase, Web of Science, Scopus, CENTRAL, PsycINFO, and CNKI. Studies were systematically screened according to the PRISMA guidelines, and the quality of the included literature was assessed using the Cochrane Risk of Bias tool. The size of the effect was expressed by means of SMD and 95% confidence interval, and subgroup analyses were performed to explore sources of heterogeneity.

**Results:**

A total of 11 RCTs (11 independent datasets) involving 750 participants were included in the analysis. The meta-analysis revealed a significant overall effect of soft Chinese martial arts on reducing depressive symptoms in college students (SMD = –0.71, 95% CI: –1.23 to –0.18, *p* = 0.008). Subgroup analyses suggested potential moderating effects of intervention duration, frequency, and session length. For example, the effect size for the 9-14 week subgroup was SMD = –1.35 (95% CI: –2.10 to –0.60) and for the 20-60 minute single session duration subgroup was SMD = –0.69 (95% CI: –1.36 to –0.03). Heterogeneity was high (*I2* = 82.6%, *p* < 0.001). Egger’s test was not statistically significant (*p* = 0.397), but publication bias cannot be ruled out due to the small number of studies.

**Conclusion:**

Soft Chinese martial arts, particularly Tai Chi and Baduanjin, may be an effective non-pharmacological intervention for reducing depressive symptoms in college students, providing preliminary evidence for mental health programs. However, results should be interpreted with caution because of high heterogeneity, risk of bias, and possible publication bias. The certainty of evidence is very low according to the GRADE framework.

**Systematic review registration:**

https://www.crd.york.ac.uk/prospero/display_record.php?RecordID=1129413, identifier CRD420251129413.

## 1 Introduction

Globally, college students face significant mental health challenges, with depressive symptoms being particularly prevalent (1). The World Health Organization (WHO) identifies depression as a leading contributor to the global burden of disease and disability. The transitional stage of higher education, characterized by heightened academic, social, and career pressures, makes college students a vulnerable population (2–4). Cross-sectional studies have reported high rates of depression, anxiety, and stress among this group, with prevalence rates for depressive symptoms ranging from 12% to over 50% in different countries (5). For example, a study in Spain found that 18.4% of university students had depressive symptoms (4), while data from Turkey indicated that 27.1% exhibited moderate to severe depression (6). In China, average anxiety levels among university students often exceed normal thresholds (2), and sedentary behaviors are strongly linked to increased stress and depression (7). The impact of depression extends beyond psychological distress, impairing academic performance, social functioning, and increasing the risk of substance abuse and suicide (2, 3). Consequently, identifying effective and sustainable interventions for depression in this population has become a critical public health priority.

Physical exercise is a well-established method for improving mental well-being, supported by extensive empirical evidence (8). Martial arts, a unique form of physical activity, integrates physical training with mental focus, philosophical principles, and self-discipline (1, 9, 10). The present review focuses on soft Chinese martial arts, specifically Tai Chi and Baduanjin, which are characterized by slow, controlled movements and mind–body integration. Unlike general exercise, martial arts emphasizes mind-body integration, focusing on the harmonious development of internal cultivation and external skills. This dual focus makes it a distinctive form of “mind-body intervention” and “meditative exercise” (1, 10). On a physiological level, martial arts training can enhance cardiovascular function, muscle strength, and flexibility, which in turn promote the release of endorphins and other neurotransmitters that induce positive emotional states (1, 11). The inclusion of meditation and breathing techniques helps to promote relaxation, reduce sympathetic nervous system activity, and regulate the hypothalamic-pituitary-adrenal (HPA) axis, all of which are associated with emotional regulation (10, 11). From a psychological perspective, martial arts training improves emotional regulation, self-efficacy, and a sense of mastery, providing additional support for alleviating depressive symptoms.

A growing body of research has demonstrated the mental health benefits of various martial arts styles, particularly Chinese martial arts. In this review, we adopt the widely used classification that distinguishes “soft” martial arts — which emphasize slow, controlled movements, breathing regulation, and mind–body integration, such as Tai Chi, Qigong, and Baduanjin — from “hard” martial arts — which emphasize forceful striking and direct physical engagement, such as karate, taekwondo, and mixed martial arts (12, 13). Tai Chi and Qigong, for instance, have been shown to effectively reduce symptoms of depression, stress, and anxiety (1, 14). A meta-analysis confirmed that Tai Chi is significantly associated with improved psychological well-being (14, 15). Similarly, Baduanjin has been found to alleviate anxiety, obsessive-compulsive symptoms, and depression (16), while also enhancing self-efficacy, mood, and sleep quality (11, 17). Even hard martial arts training has been shown to positively impact mood and reduce the risk of depression (16). Chinese martial arts can generally enhance overall psychological well-being and quality of life (13, 18–21). At a social level, martial arts can foster a sense of belonging and provide social support, which is beneficial for alleviating the loneliness and social isolation often associated with depression (10). Meanwhile, as a form of physical activity, martial arts can also effectively address the current widespread sedentary behavior pattern (22).

In the realm of mental health research, a growing body of systematic reviews and meta-analyses has explored the effects of physical activity and mind-body interventions on psychological outcomes. These syntheses consistently show that various forms of exercise, including aerobic activities, yoga, and Tai Chi, are effective in reducing symptoms of depression, anxiety, and stress across diverse populations (1, 23, 24). However, these broader reviews often have limitations concerning their specific focus on college students and the nuanced effects of different martial arts styles on depressive mood. For example, most existing empirical studies have small sample sizes or are confined to specific martial arts disciplines; while Tai Chi is the most extensively researched form, the mental health benefits of other styles, such as hard martial arts, have been relatively understudied (1). Additionally, a significant portion of the current literature combines martial arts interventions with other therapeutic approaches, such as pharmacotherapy or psychotherapy, which makes it challenging to precisely isolate the unique effects attributable to martial arts alone (25, 26).

It is also noteworthy that a low proportion of martial arts intervention studies have focused on the adult population aged 18–65, with the majority of research concentrating on children, adolescents, and the elderly (25). Furthermore, inconsistent findings exist within the literature; for instance, one study on a Baduanjin intervention in college students did not demonstrate significant improvements in mental health (27), a result that might be linked to the high-intensity physical activity concurrently undertaken by the control group. These methodological limitations and heterogeneous outcomes have substantially hindered the promotion and clinical application of martial arts as a standardized and effective intervention.

This study aims to address this critical gap by providing a comprehensive and quantitative synthesis of the existing evidence. The specific objectives are: (1) to systematically evaluate the overall effect of soft Chinese martial arts interventions on depressive mood in college students; (2) to perform a quantitative analysis to provide evidence-based support for soft Chinese martial arts as a non-pharmacological intervention; and (3) to explore potential moderator variables, including the type of martial art, intervention duration, frequency, and session length. The findings are expected to deepen our understanding of the mechanisms by which soft Chinese martial arts affect mental health and provide practical guidance for developing mental health strategies for college students.

However, it is important to recognize that the psychological benefits of martial arts practice are not inherent or guaranteed. Rather, they are likely to be moderated by contextual and pedagogical factors, including the quality of instruction, the safety and ethical climate of the training environment, the selection of training partners, the progressive adaptation of practice tasks, and the regulation of training intensity (13, 20, 21). These considerations underscore that martial arts should not be viewed as a uniform intervention category, but as a set of practices whose mental health effects may vary according to how, where, by whom, and with whom they are delivered—a perspective that informs the interpretation of the findings reported below.

## 2 Materials and methods

### 2.1 Study registration

This systematic review and meta-analysis was designed and conducted following the Preferred Reporting Items for Systematic Reviews and Meta-Analyses (PRISMA) guidelines (28). This study protocol was registered with PROSPERO (International Prospective Register of Systematic Reviews) with registration number CRD420251129413.

### 2.2 Search strategy and databases

A comprehensive and systematic literature search was performed across seven electronic databases, with the search completed by July 31, 2025. The databases included: PubMed/MEDLINE, Embase, Web of Science (Core Collection), Scopus, Cochrane Central Register of Controlled Trials (CENTRAL), PsycINFO, and China National Knowledge Infrastructure (CNKI) for Chinese literature. The search strategy was developed in consultation with an experienced librarian, utilizing a combination of keywords related to martial arts, college students, and depressive mood. Boolean operators (AND, OR) were employed to combine terms effectively. Grey literature sources and trial registries were not systematically searched, as this was not specified in our PROSPERO protocol; this limitation is acknowledged in the Discussion (section 4.2).

The key search terms included, but were not limited to:

1. Martial Arts: (“martial arts” OR “kung fu” OR “wushu” OR “tai chi” OR “taijiquan” OR “qigong” OR “baduanjin” OR “mixed martial arts”).
2. College Students: (“college students” OR “university students” OR “undergraduate” OR “postgraduate” OR “higher education”).
3. Depressive Mood: (“depression” OR “depressive symptoms” OR “depressive mood” OR “mood disorder” OR “mental health” OR “psychological distress”).

The search was iterative, refining terms as relevant articles were identified. Reference lists of included studies and relevant systematic reviews were also manually screened to identify any additional eligible articles not captured by the initial database searches. Complete search strategies for each database, including database-specific syntax, field tags, date limits, language limits, and Chinese search terms, are provided in **Supplementary Table 1**.

To maximize search sensitivity and minimize the risk of missing eligible studies, we deliberately used broad search terms (e.g., “martial arts”) rather than the narrower term “soft Chinese martial arts” in the search strategy. The latter is not suitable as a search term, because primary studies typically report only the specific intervention name (e.g., “Tai Chi” or “Baduanjin”) and rarely use this overarching term in their titles or abstracts. Accordingly, the classification of the included studies as soft Chinese martial arts was determined a posteriori during study screening and data extraction, based on the specific interventions actually delivered in the included trials. This approach is consistent with standard methodological guidance, which recommends prioritizing search sensitivity and basing *post-hoc* intervention classifications on the interventions actually delivered in the primary studies.

### 2.3 Eligibility criteria

Studies were included in this meta-analysis if they met the following pre-defined PICOS criteria:

P (Population): College or university students (undergraduate or postgraduate) aged 18 years and above. Studies focusing on specific clinical populations (e.g., diagnosed major depressive disorder) were included if the participants were university students.

I (Intervention): Any form of martial arts intervention (e.g., Tai Chi, Qigong, Baduanjin, mixed martial arts) delivered as a structured program. The intervention must explicitly involve martial arts training, regardless of whether it was combined with other non-pharmacological interventions (e.g., health education, relaxation techniques) as long as martial arts was a primary component.

C (Comparator): Control groups could include usual care, no intervention, waiting list, passive controls (e.g., educational lectures), or active controls (e.g., other forms of exercise, recreational activities).

O (Outcome): Must report quantitative data on depressive mood or symptoms. Acceptable outcome measures included validated self-report questionnaires or clinician-rated scales.

S (Study Design): Only randomized controlled trials (RCTs) were eligible for inclusion. Only full-text articles published in English or Chinese were considered.

Studies were excluded if they were:

1. Review articles, meta-analyses, conference abstracts, protocols, book chapters, or qualitative studies. Master's theses retrieved from CNKI were included if they provided full-text, retrievable data and had undergone formal thesis committee review, as they represent primary research with original data. Their inclusion was also intended to reduce the risk of publication bias, because dissertations are generally less prone than peer-reviewed journal articles to the selective reporting of positive results. To ensure full transparency regarding study type, all included dissertations are clearly marked with a dagger symbol (†) in **Table 1**, with a footnote at the table’s base explaining that † denotes a dissertation, and are designated as master's theses ([master's thesis]) in the reference list.
2. Focused solely on physical health outcomes without reporting on depressive mood.
3. Involved participants with severe mental illness (e.g., schizophrenia, bipolar disorder, severe clinical depression requiring hospitalization) where depressive mood was a secondary symptom of a primary psychotic disorder.
4. Interventions that involved martial arts purely for competitive performance with no explicit health promotion aim.

**Table 1:** Basic information of included literature.

First Author	Year	Sample (I/C)	Age (V/C, mean ± SD or range)	Sex distribution	Intervention	Control type	Duration (wks)	Freq (s/wk)	Session (min)	Baseline depression (I, mean ± SD)	Baseline depression (C, mean ± SD)	Scale
Li C (29)	2014	20/20	20-23/NR	NR	Baduanjin	Passive control	45	5	60	55.60 ± 4.12	54.70 ± 3.96	SDS
Yang Y† (30)	2021	20/20	18-20/18-20	all female	Tai Chi	Passive control	8	2	45-60	50.95 ± 7.00	58.50 ± 5.39	SDS
Mao H (31)	2008	52/52	18-20/18-20	all female	Tai Chi	Passive control	18	3	60	33.25 ± 6.08	37.00 ± 7.01	SDS
Zhang J (32)	2023	9/9	24.2 ± 4.07/22.5 ± 5.95	NR	Bafa Wubu Tai Chi	Passive control	8	5	60	43.39 ± 10.17	51.80 ± 12.16	SDS
Su Y† (33)	2010	15/15	NR/NR	all female	24-form Tai Chi	Passive control	13	2	80	17.27 ± 5.69	20.27 ± 6.98	BDI
Huang B† (34)	2008	32/32	18.6/18.4	all female	Simplified 24-form Tai Chi	Active control	14	1	90	7.12 ± 5.59	10.55 ± 5.28	BDI
Su Y (merged) (35)	2013	24/26	NR/NR	mixed (vast NR)	Simplified Tai Chi	Passive control	12	2/5	60	see note^a^	see note^a^	BDI
Ji X (36)	2008	127/118	18/NR	NR	Tai Chi	Passive control	20	1-5	50	1.45 ± 0.37	1.63 ± 0.58	SCL-90
Sun Y (37)	2022	30/30	22.2 ± 1.5/22 ± 1.5	NR	Tai Chi	Passive control	17	2	90	1.34 ± 0.36	1.32 ± 0.50	SCL-90
Xiong G (38)	2010	12/14	NR/NR	NR	Tai Chi	Passive control	16	3	50	1.94 ± 0.18	1.68 ± 0.17	SCL-90
Zhang Y (39)	2023	34/39	19.23 ± 0.98/19.16 ± 1.05	NR	Baduanjin	Passive control	12	3	60	0.52 ± 0.32	0.85 ± 0.38	SCL-90

^†^Master's theses retrieved from CNKI that underwent formal thesis committee review; the other eight entries are peer-reviewed journal articles.

I, intervention group; C, control group; SD, standard deviation; NR, not reported; SDS, Self-Rating Depression Scale; BDI, Beck Depression Inventory; SCL-90, Symptom Checklist-90.

Su 2013 (35) (merged): Baseline BDI scores ranged 9.47–18.24 (intervention) and 26.57–28.83 (control). See original for arm-level data.

All studies reported immediate post-intervention outcomes. Intervention setting, adherence, and adverse events were insufficiently reported in the primary studies.

### 2.4 Study selection and data extraction

Two independent reviewers independently screened titles and abstracts retrieved from the databases based on the eligibility criteria. Any discrepancies were resolved through discussion or, if necessary, by consulting a third reviewer. Full texts of potentially eligible articles were then retrieved and independently reviewed by the same two reviewers to confirm final inclusion. The reasons for exclusion were documented at the full-text review stage.

A standardized data extraction form, developed prior to data extraction, was used by two independent reviewers to extract relevant data from each included study. Extracted data included:

1. Study Characteristics: First author, publication year, study design, sample size (intervention and control groups), participants’ demographics (age, gender distribution), and inclusion/exclusion criteria.
2. Intervention Characteristics: Type of martial art, specific techniques/style, intervention duration (weeks), frequency (sessions per week), session length (minutes).
3. Comparator Characteristics: Type of control group (e.g., passive, active, usual care).
4. Outcome Data: Depressive mood assessment tool used, mean scores and standard deviations (or other statistics convertible to these, such as median/interquartile range if means were unavailable) for depressive mood at baseline and post-intervention for both intervention and control groups.
5. Risk of Bias Information: Details necessary for assessing the risk of bias for each study.
6. Discrepancies during data extraction were resolved by discussion between the two reviewers or by consultation with a third reviewer.

### 2.5 Risk of bias assessment

The methodological quality and risk of bias for each included study were independently assessed by two reviewers. For Randomized Controlled Trials (RCTs), the Cochrane Risk of Bias tool was utilized. For randomized controlled trials, the Cochrane Risk of Bias tool was used to assess seven domains: random sequence generation, allocation concealment, blinding of participants and personnel, blinding of outcome assessment, incomplete outcome data, selective reporting, and other bias. Each domain was judged as having a low, unclear, or high risk of bias. Disagreements were resolved through discussion or by a third reviewer.

### 2.6 Data synthesis and statistical analysis

A meta-analysis was performed using Stata 15 and Review Manager 5.4. For continuous outcomes (depressive mood scores), standardized mean differences (SMDs) and their 95% confidence intervals (CIs) were calculated to represent the effect size. Effect sizes were derived from post-intervention scores. To correct for small sample bias, Hedges’ g was used as the effect size measure. Because different studies used different depression scales (SDS, BDI, SCL-90), we used SMD to harmonize effect sizes across studies. SMDs of 0.2, 0.5, and 0.8 were considered small, moderate, and large effects, respectively.

Statistical heterogeneity among studies was assessed using the chi-square test (*I*^2^) and the Tau-squared (*τ*^2^) statistic. *I*^2^ values of 25%, 50%, and 75% were indicative of low, moderate, and high heterogeneity, respectively. A random-effects model with restricted maximum likelihood (REML) estimation was chosen for data synthesis given the expected heterogeneity across diverse intervention types, durations, and populations. For studies that did not report standard deviations, we attempted to impute them from available statistics (e.g., confidence intervals, t-values, p-values) where possible; otherwise, they were excluded from the analysis.

First, we selected post-intervention scores rather than change scores as the primary effect metric for three reasons. Because the computation of change scores requires complete baseline data together with a baseline-to-follow-up correlation coefficient, and because several included studies did not report these statistics in full, change scores could not be computed consistently across all trials. Change scores are also more sensitive to regression to the mean, which may inflate the estimated effect when participants are selected on the basis of elevated baseline scores. By contrast, post-intervention scores analyzed with a random-effects model represent the standard approach for synthesizing evidence from multicenter and heterogeneous interventions and, therefore, yield results with greater generalizability.

Second, we assessed baseline comparability between the intervention and control groups using a descriptive approach rather than formal statistical testing, because most primary studies did not report the complete statistics (e.g., baseline SDs and between-group p-values) required for formal tests. This descriptive assessment identified between-group differences in baseline depressive scores in a minority of the included studies; because these differences were not consistently reported or formally tested in the primary studies, their potential influence on the pooled estimates cannot be excluded and is addressed in the Limitations.

Third, for studies that did not report standard deviations (SDs), we first attempted to impute them from available statistics (e.g., confidence intervals, t-values, or p-values) using standard formulas, because such imputation maximizes the inclusion of eligible data; studies for which SDs could not be reliably imputed were excluded from the meta-analysis.

Subgroup analyses were conducted according to intervention duration (1–8, 9–14, and ≥15 weeks), weekly frequency (1–2 and 3–5 sessions/week), session duration (20–60 and >60 min), and control type (active and passive). Subgroup analysis by martial arts type was not conducted because all eligible studies examined soft Chinese martial arts, and no hard martial arts studies met the inclusion criteria.

Publication bias was assessed using Egger’s regression test and visually inspected using funnel plots. Due to the small number of studies (k = 11), the power of Egger’s test is limited; therefore, results were interpreted conservatively.

## 3 Results

### 3.1 Flow of study selection

The initial comprehensive search across seven electronic databases yielded a total of 13,135 records. After removing 185 duplicate entries, 12,950 unique records were subjected to title and abstract screening. Following a meticulous screening of titles and abstracts against pre-defined inclusion and exclusion criteria, 12,863 records were excluded, leaving 87 reports that were sought for retrieval. Of these, 30 reports could not be retrieved. The remaining 57 reports were assessed for eligibility through full-text review, and 46 were excluded due to reasons such as non-compliance with experimental design requirements or incongruent outcome measures. Consequently, 11 randomized controlled trials (comprising 11 independent data sets) were ultimately included for the meta-analysis. The detailed flow of the study selection process is illustrated in the PRISMA flow diagram (**Figure 1**).

**Figure 1:**
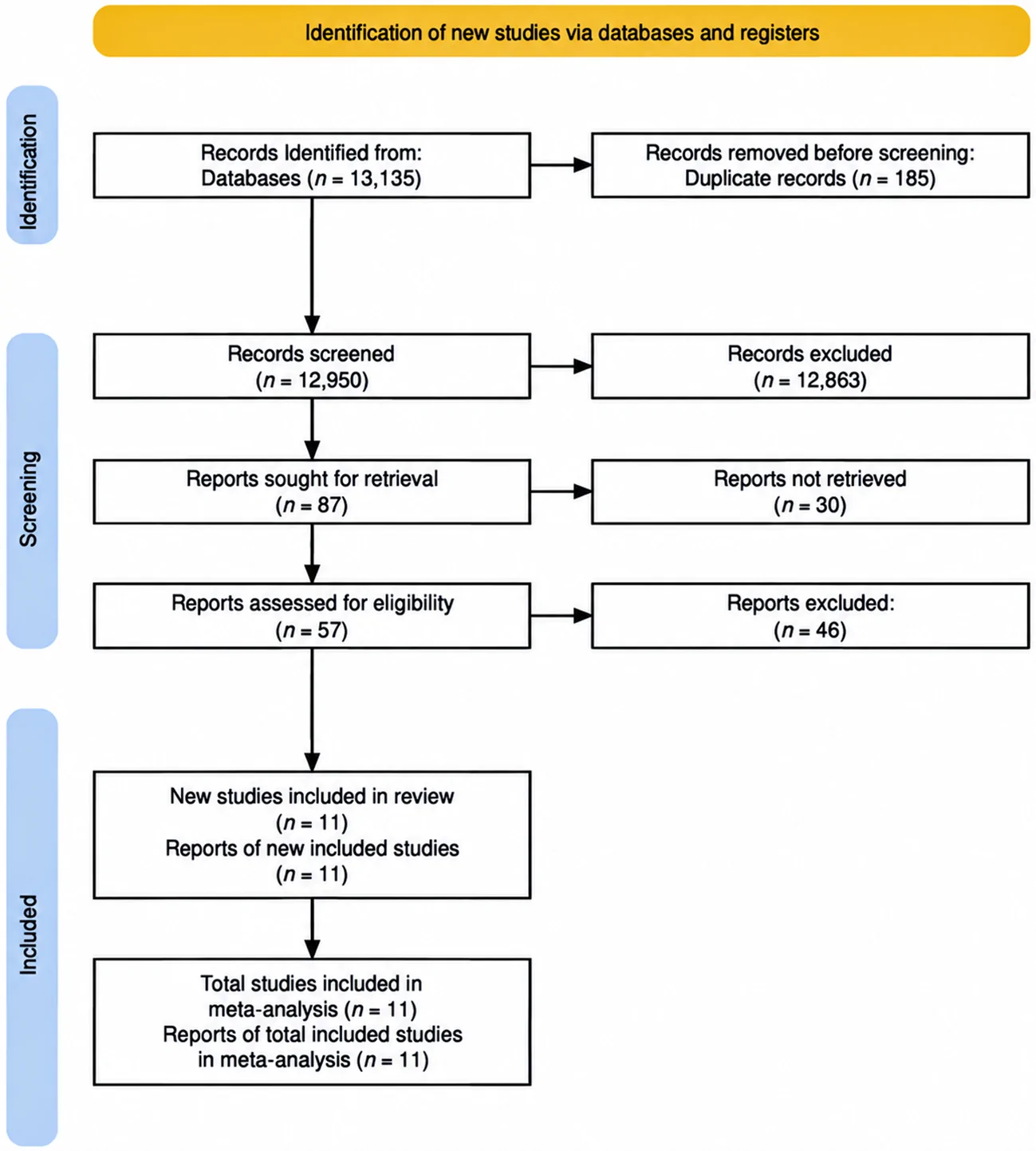
PRISMA 2020 flow diagram.

### 3.2 Characteristics of included studies

A total of 11 articles (11 independent data results) were included in this study, and all were randomized controlled trials. Among them, 375 were in the experimental group and 375 in the control group. Zung’s depression scale/Self-rating Depression Scale (SDS), Beck Depression Inventory (BDI) and psychological symptom Checklist 90 (SCL-90) were used as the evaluation indicators. The basic information of the included literature is shown in **Table 1**. All included studies examined soft martial arts (Tai Chi and Baduanjin). No studies on hard martial arts (e.g., karate, taekwondo, mixed martial arts) met the inclusion criteria; therefore, comparison by martial arts type was not possible. Of the 11 included studies, eight were peer-reviewed journal articles and three were master's theses (Yang 2021 (30), Su 2010 (33), Huang 2008 (34)) retrieved from CNKI that had undergone formal thesis committee review; these three dissertations are now explicitly marked with a dagger symbol (†) in **Table 1**, with a footnote at the table’s base explaining that † denotes a dissertation, and are also marked as master's theses ([master's thesis]) in the reference list (see Refs 30, 33, 34), ensuring full transparency regarding study type.

### 3.3 Risk of bias assessment results

The methodological quality of the 11 included studies was assessed using the Cochrane Risk of Bias tool (**Figure 2**). Most studies had low risk of bias for random sequence generation, incomplete outcome data, selective reporting, and other biases. However, allocation concealment was unclear in 10 out of 11 studies (91%), and blinding of participants and personnel was unclear or high risk in 8 out of 11 studies (73%). Blinding of outcome assessment was also unclear in 9 out of 11 studies (82%). Overall, the risk of bias across studies was moderate to high, primarily due to inadequate reporting of allocation concealment and blinding procedures, which is common in exercise-based intervention trials. These limitations should be considered when interpreting the findings.

**Figure 2:**
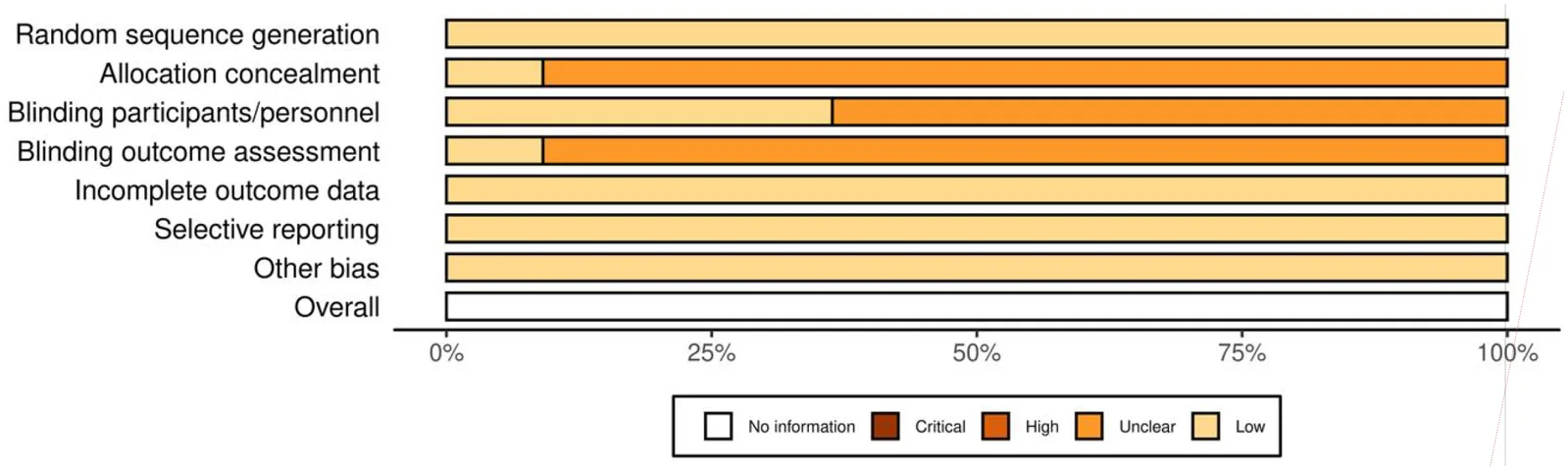
Schematic diagram of risk bias.

### 3.4 Meta-analysis results

A meta-analysis was conducted on the data from the 11 independent studies (after correcting the unit-of-analysis error). The results showed substantial heterogeneity among the studies (χ^2^ = 57.56, df = 10, *p* < 0.001; I^2^ = 82.6%). A random-effects model was therefore used. The pooled effect was SMD = –0.71 (95% CI: –1.23 to –0.18). This result indicates that, compared with control groups (passive or active), soft Chinese martial arts interventions may significantly reduce depressive symptoms in college students. The forest plot is presented in **Figure 3**.

**Figure 3:**
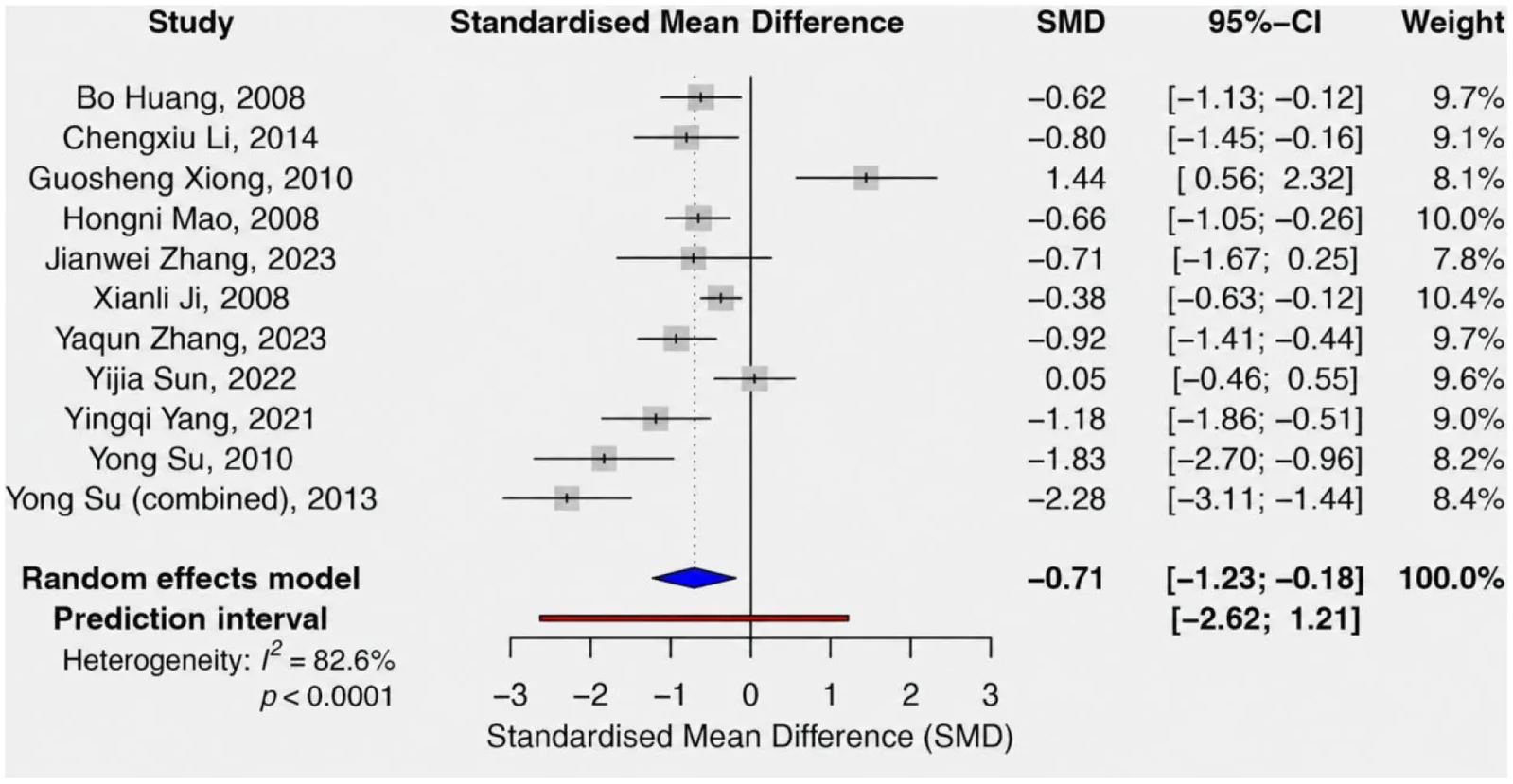
Meta-analysis forest plot.

#### 3.4.1 Sensitivity analysis

A leave-one-out sensitivity analysis was performed to assess the influence of each individual study on the pooled estimate. After sequentially excluding each study, the pooled SMD ranged from –0.86 (95% CI: –1.25 to –0.48) to –0.57 (95% CI: –1.04 to –0.09), and all remained statistically significant (all *p* < 0.05). Heterogeneity remained high across iterations (I^2^ range 75.5%–84.4%). No single study dominated the overall estimate; however, heterogeneity remained high across all iterations, and the findings should be interpreted with caution given the very low certainty of evidence.

A separate sensitivity analysis was conducted excluding the three master's theses (Yang, 2021 (30), Su, 2010 (33), Huang, 2008 (34)) to assess whether the inclusion of non-peer-reviewed studies influenced the overall effect. After excluding these dissertations, the pooled effect from the remaining 8 studies remained statistically significant (SMD = –0.68, 95% CI: -1.25 to -0.11, p = 0.019), with persistently high heterogeneity (I^2^ = 84.2%). These results indicate that the overall conclusion is not driven by the inclusion of dissertations.

#### 3.4.2 Period of intervention

Subgroup analyses were exploratory in nature and should be interpreted as hypothesis-generating only.

When grouped by intervention duration (**Figure 4**), programs lasting 1-8 weeks (2 studies) yielded a significant effect (SMD = –1.03, 95% CI: –1.58 to –0.48, *p* < 0.001), with no evidence of heterogeneity (*I^2^* = 0%, Q = 0.61, df = 1, *p* = 0.433). For interventions of 9-14 weeks (4 studies), the pooled SMD was –1.35 (95% CI: –2.10 to –0.60, *p* < 0.001), but heterogeneity was substantial (*I^2^* = 79.0%, Q = 14.31, df = 3, *p* = 0.0025). In contrast, trials lasting ≥15 weeks (5 studies) did not show a statistically significant benefit (SMD = –0.13, 95% CI: –0.83 to 0.58, *p* = 0.729), despite high heterogeneity (*I^2^* = 82.2%, Q = 22.51, df = 4, *p* < 0.001). Thus, shorter interventions (≤14 weeks) appeared effective, whereas longer ones (≥15 weeks) did not.

**Figure 4:**
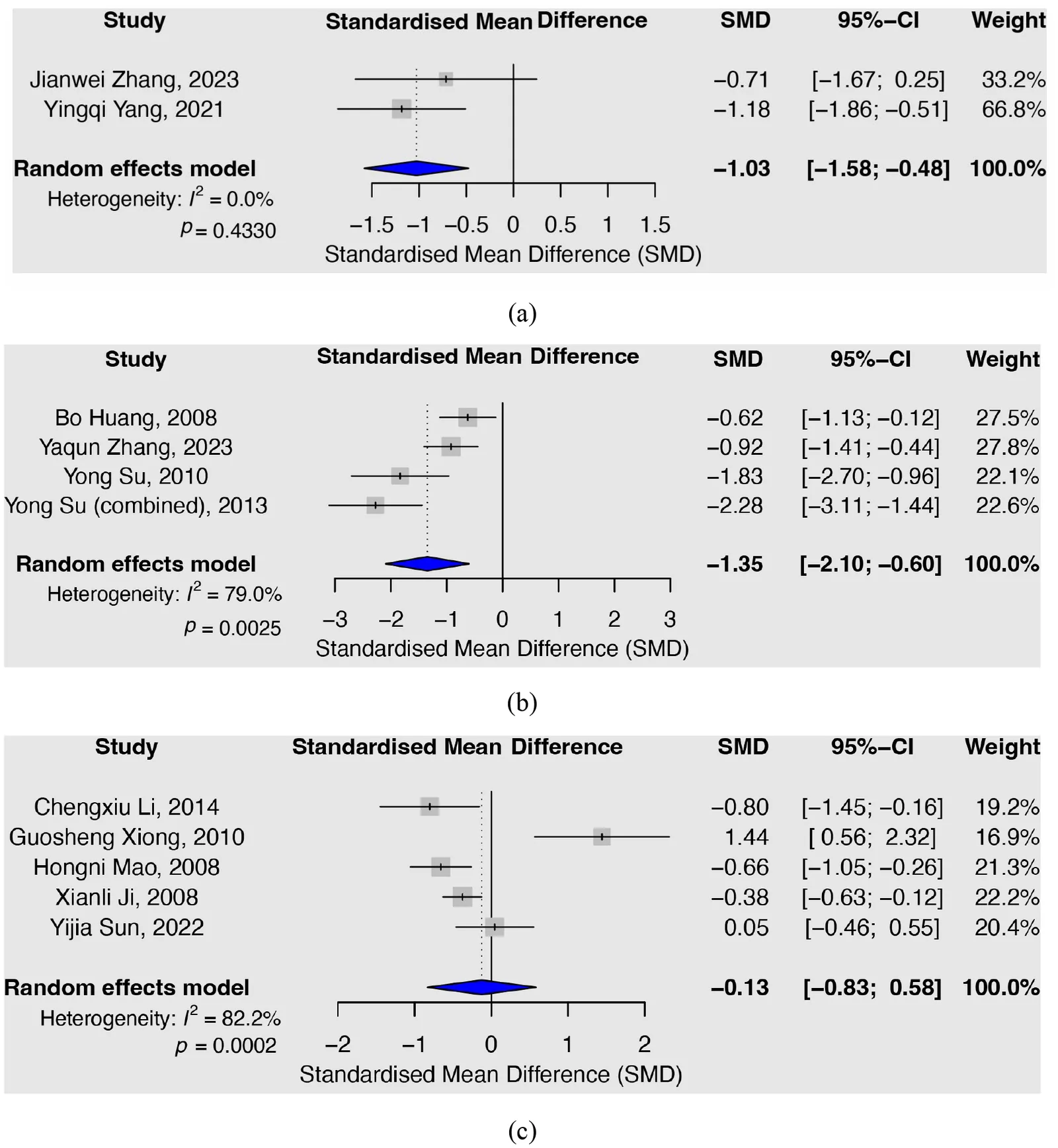
Forest plot of subgroup analysis during the intervention period. **(a)** Forest plot with intervention period of 1–8 weeks. **(b)** Forest plot with intervention period of 9–14 weeks. **(c)** Intervention periods of 15 weeks or more.

#### 3.4.3 Frequency of intervention

A separate analysis by weekly session frequency (**Figure 5**) revealed that interventions delivering 1-2 sessions per week (5 studies) significantly reduced depressive symptoms (SMD = –1.13, 95% CI: –1.94 to –0.31, *p* = 0.007). However, heterogeneity was very high (*I^2^* = 86.6%, Q = 29.83, df = 4, *p* < 0.001). For interventions with 3-5 sessions per week (6 studies), the pooled estimate was not significant (SMD = –0.38, 95% CI: –1.02 to 0.26, *p* = 0.241), also accompanied by considerable heterogeneity (*I^2^* = 79.3%, Q = 24.15, df = 5, *p* < 0.001). Given the high heterogeneity in both subgroups, the influence of weekly frequency on the intervention effect remains uncertain.

**Figure 5:**
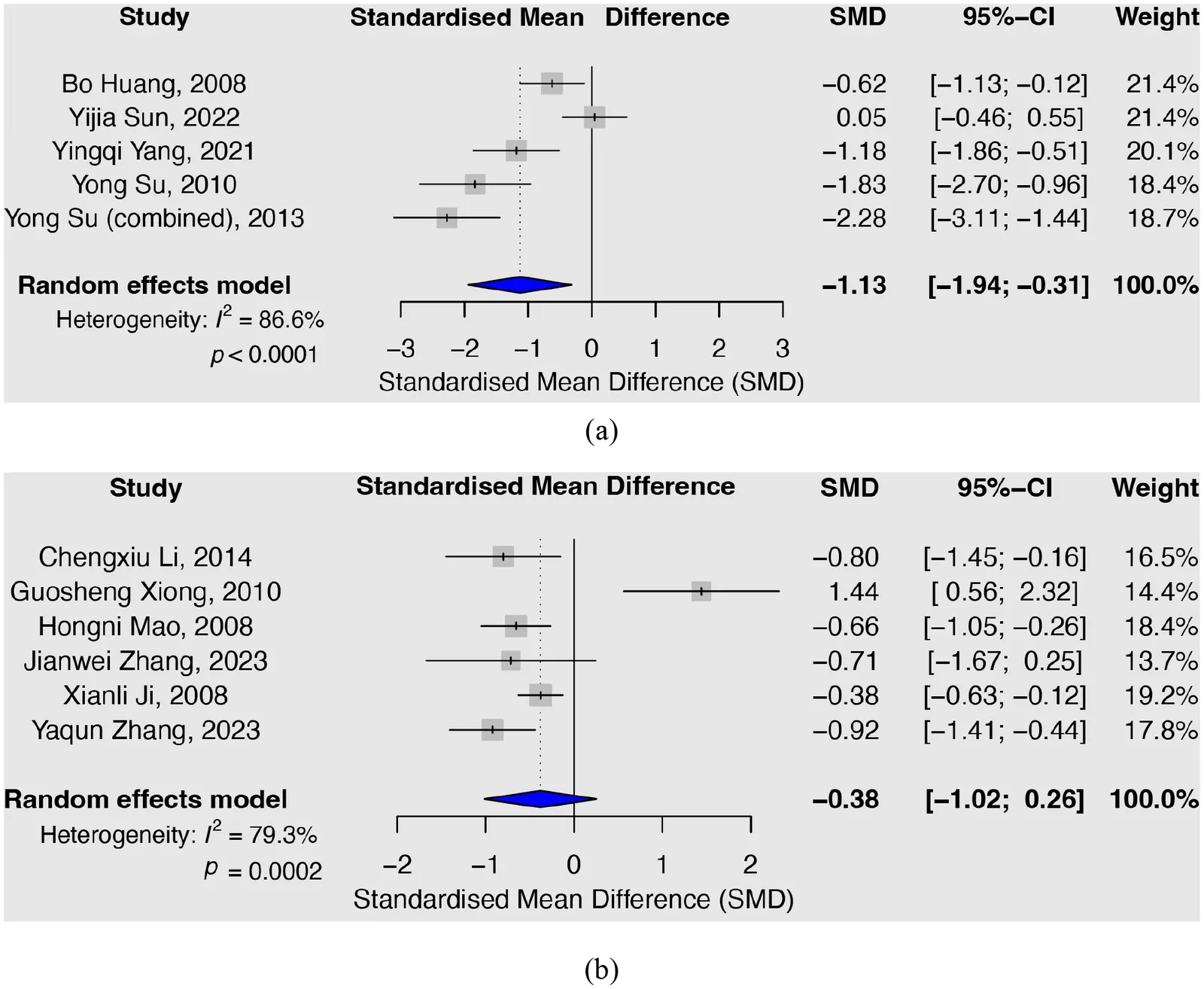
Forest plot of subgroup analysis of intervention frequency. **(a)** The frequency of intervention was 1–2 times per week. **(b)** The frequency of intervention was 3–5 times per week.

#### 3.4.4 Duration of a single intervention

Considering the length of each session (**Figure 6**), interventions lasting 20-60 minutes (8 studies) led to a significant reduction in depressive symptoms (SMD = –0.69, 95% CI: –1.36 to –0.03, *p* = 0.041), albeit with high heterogeneity (*I^2^* = 84.2%, Q = 44.29, df = 7, *p* < 0.001). In contrast, session lengths exceeding 60 minutes (3 studies) produced a non-significant pooled effect (SMD = –0.75, 95% CI: –1.79 to 0.28, *p* = 0.154), again with high heterogeneity (*I^2^* = 85.4%, Q = 13.70, df = 2, *p* = 0.001). These findings suggest that sessions of 20-60 minutes may be beneficial, whereas longer sessions did not demonstrate a clear advantage, possibly owing to the limited number of studies or lower adherence.

**Figure 6:**
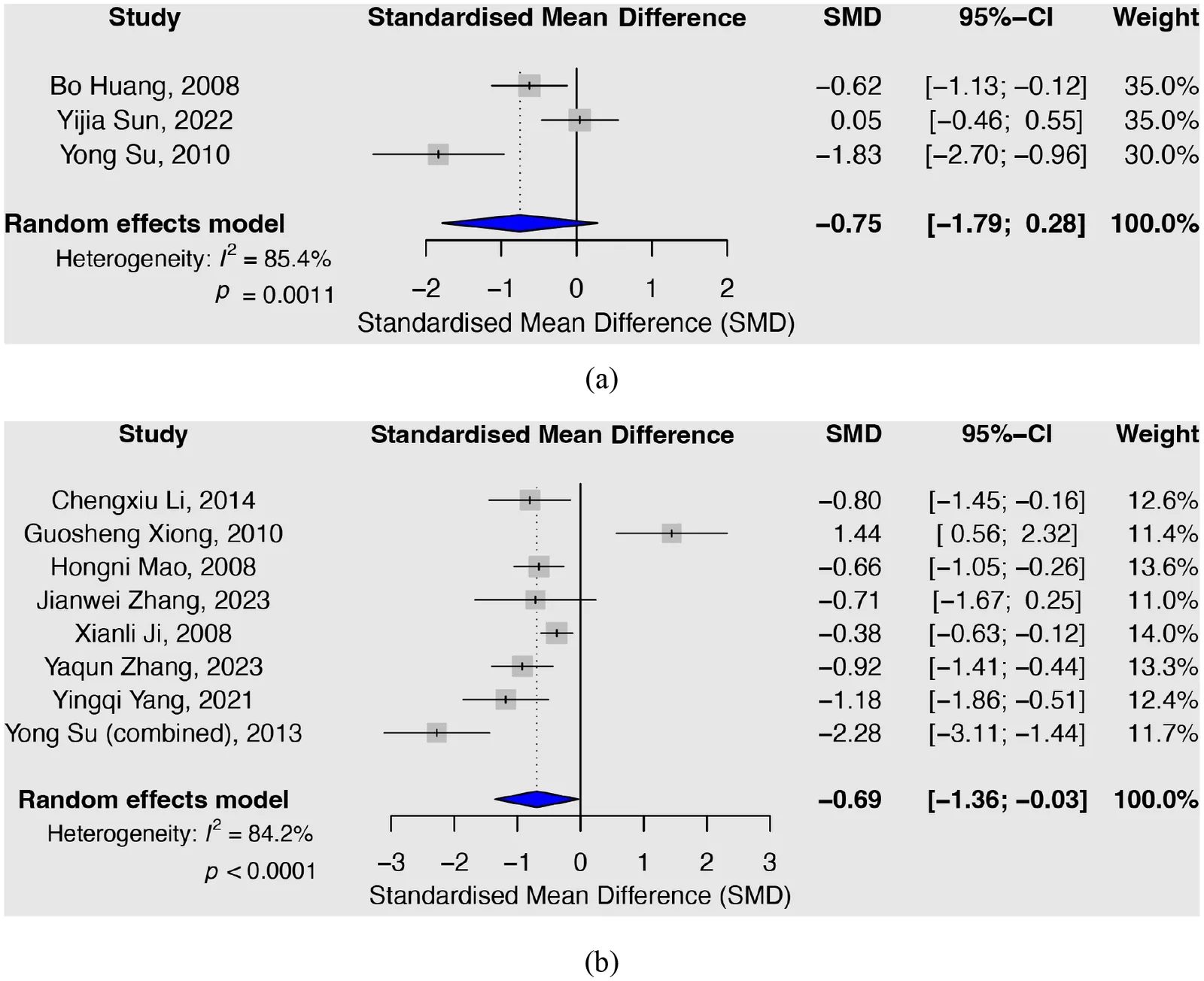
Forest plot of subgroup analysis for duration of single intervention. **(a)** Forest plot with a single intervention duration of 20–60 minutes. **(b)** The duration of a single intervention is more than 60 minutes.

#### 3.4.5 Type of control group

Only one study (Huang, 2008 (34)) used an active control condition, in which participants engaged in other physical education activities—including aerobics, table tennis, volleyball, badminton, basketball, and artistic gymnastics—rather than Tai Chi. Its effect was SMD = –0.62 (95% CI: –1.13 to –0.12). The remaining 10 studies used passive controls (no intervention or usual care), with a pooled SMD of –0.74 (95% CI: –1.29 to –0.19). Because the active-control subgroup contained only one study, no reliable conclusion can be drawn regarding differences between active and passive control conditions.

#### 3.4.6 Publication bias

Egger’s linear regression test was performed to assess publication bias. The test did not reach statistical significance (t = –0.89, df = 9, *p* = 0.397). The Egger's regression plot is shown in **Figure 7**. However, with only 11 studies included, the test has limited statistical power. Therefore, publication bias cannot be ruled out.

**Figure 7:**
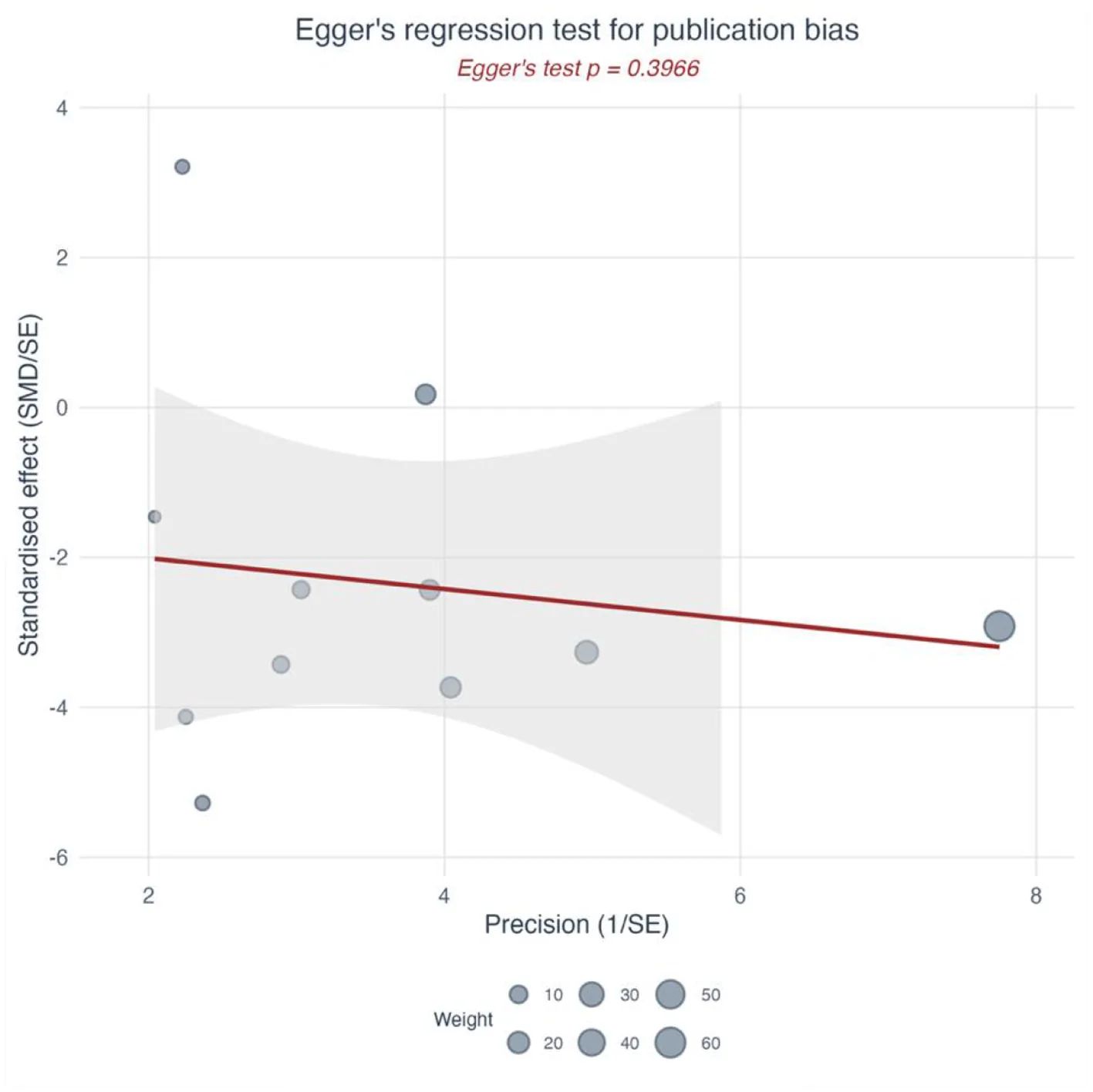
Publication bias.

## 4 Discussion

### 4.1 Summary and interpretation of findings

This meta-analysis provides preliminary evidence of lower post-intervention depressive symptom scores among college students receiving soft Chinese martial arts interventions than among those in the control groups. The magnitude of the pooled estimate was moderate-to-large, but its interpretation is limited by substantial heterogeneity and very low certainty of evidence. The pooled effect size of SMD = –0.71 is statistically significant but preliminary and suggests that soft Chinese martial arts practice can serve as a potentially effective non-pharmacological intervention for depression in this population. This finding is consistent with the broader literature on the mental health benefits of exercise but offers a more nuanced understanding by focusing specifically on the unique attributes of martial arts (40–42). Our results are also in line with a recent systematic review by Ciaccioni et al. (2024) (12), which highlighted the high heterogeneity and the limited evidence for mental health benefits of martial arts and combat sports in adults, further underscoring the need for cautious interpretation.

While a number of studies have demonstrated the mental health benefits of exercise (43, 44), our research offers a more focused understanding by concentrating specifically on martial arts interventions for college students. The positive effect we found is consistent with earlier findings on the benefits of Tai Chi and Qigong across diverse populations (24) and provides preliminary evidence extending their potential effectiveness to the subgroup of college students. This finding provides preliminary but not conclusive evidence regarding the potential of soft Chinese martial arts as a mental health intervention for this demographic.

The exploratory subgroup analyses identified possible variations in effect estimates across intervention duration, frequency, and session-length categories. Our findings indicate that the intervention effect is most pronounced with specific durations, frequencies, and session lengths. The non-significant effects for interventions lasting more than 15 weeks or for single sessions exceeding 60 minutes may be attributed to a lack of statistical power in these subgroups or a potential “critical dose” effect. It is plausible that an optimal dose exists for participants to achieve the unique “mind-body integration” state inherent to soft Chinese martial arts, thereby maximizing the meditative and therapeutic benefits beyond mere physical exertion.

Subgroup analyses further suggested that the beneficial effect was most pronounced for interventions lasting 1-8 weeks or 9-14 weeks, delivered 1-2 times per week, and with single sessions of 20-60 minutes, whereas longer interventions (≥15 weeks) and longer single sessions (>60 minutes) yielded non-significant effects, likely owing to the limited sample sizes in these subgroups. These patterns suggest the presence of potential moderating effects of intervention parameters, which may enable participants to fully engage in the unique mind-body integration state characteristic of soft Chinese martial arts, thereby activating deep-seated meditative and relaxation mechanisms. Given the exploratory nature of the subgroup analyses and the small numbers of studies in some subgroups, however, these findings should be interpreted with caution.

From a neurobiological perspective, we speculate on a potential mechanistic pathway through which soft Chinese martial arts might exert antidepressant effects, but these hypotheses require direct testing in future studies. A key neurobiological characteristic of depression is hippocampal atrophy and compromised neuroplasticity (45, 46). As a complex form of motor and cognitive training (e.g., memorizing forms, precise coordinated movements), soft Chinese martial arts might upregulate the expression of neurotrophic factors like brain-derived neurotrophic factor (BDNF) (47). This, in turn, could promote hippocampal neurogenesis and synaptic plasticity (48–50), potentially reversing brain structural changes associated with depression. A longer practice duration may reach a “critical dose” sufficient to induce sustained BDNF expression and neurogenesis (51, 52), which aligns with the observed improvements in mood.

Beyond the brain mechanisms discussed, the antidepressant effects of soft Chinese martial arts may be mediated by multiple synergistic factors. Physiologically, prolonged soft Chinese martial arts practice can effectively regulate the hypothalamic-pituitary-adrenal (HPA) axis, reducing stress hormones like cortisol and improving autonomic nervous system balance (53). Psychologically, through meditation and breath control, soft Chinese martial arts provides participants with a non-pharmacological tool for emotional catharsis and stress management (1, 24, 54). Socially, soft Chinese martial arts communities offer a structured environment that enhances an individual’s sense of belonging and provides social support, both of which are crucial psychological buffers against depression (19). The large overall effect size observed in this study may reflect the combined action of these multiple mechanisms, although this interpretation remains speculative.

While our findings suggest a positive overall effect, we note that martial arts practice may not be uniformly beneficial for depressive symptoms across all contexts and populations. Positive psychological outcomes likely depend on several contextual and pedagogical factors, including: (1) appropriate instruction by qualified coaches or instructors; (2) safeguarding procedures to ensure participant safety; (3) an ethical and supportive training environment; (4) suitable partner selection; (5) progressive task adaptation matched to individual capacity; and (6) careful regulation of training intensity. The role of coaches or instructors, peer relationships, interpersonal climate, and training culture should be considered as potential moderators of treatment effects. This perspective is supported by recent research on combat sports and wellbeing, which highlights that mental health outcomes are shaped by how, where, by whom, and with whom martial arts are practiced (13, 20, 21). Future studies should systematically examine how these contextual factors influence the mental health outcomes of martial arts interventions.

The stability of the pooled estimate was examined using sensitivity analysis. The publication bias analysis, using Egger’s test, showed a p-value of 0.397 (p > 0.05), indicating no significant publication bias.

In summary, this meta-analysis provides preliminary evidence for the overall positive effects of soft Chinese martial arts on improving depressive mood in college students. Subgroup analyses suggest that intervention duration, frequency, and session length may play a role in modulating its effectiveness, although these findings are exploratory and require confirmation. These findings offer new insights into the potential moderating effects of soft Chinese martial arts interventions and suggest that forms such as Tai Chi and Baduanjin may operate through multiple neurobiological mechanisms, including neurogenesis and neural circuit regulation. These results provide initial support for the development and promotion of evidence-based, martial arts-driven mental health interventions for college students and provide valuable directions for future research.

These findings must be interpreted in light of the very low certainty of evidence (GRADE), high heterogeneity (I^2^ = 82.6%), unclear risk of bias, reliance on self-report scales, limited active-control evidence, and possible publication bias. Given that the GRADE certainty of evidence was rated as very low, all findings — including subgroup results — should be considered preliminary and subject to change as further evidence accumulates.

### 4.2 Strengths and limitations

The primary strengths of this meta-analysis lie in its rigorous methodological design and precise focus, aimed at addressing a key gap in the existing literature. First, the study strictly followed PRISMA guidelines and was registered with PROSPERO, ensuring systematicity, transparency, and reproducibility, which reflects a high-quality standard for similar research. Second, this study specifically focused on college students, a unique and vulnerable population, and quantitatively assessed the overall effect of soft Chinese martial arts as a specific intervention. This approach provides preliminary evidence for this specific demographic, compensating for the lack of generalizability in previous research regarding both the population and the intervention measures.

Furthermore, by conducting rigorous subgroup analyses, this study explored the moderating effects of intervention period, frequency, and session length through subgroup analyses. The finding that interventions lasting 1–8 weeks and 9–14 weeks, with a frequency of 1–2 times per week, and a single session duration of 20–60 minutes yielded more significant positive effects offers preliminary, hypothesis-generating guidance for developing and implementing soft Chinese martial arts-based interventions for college students, thereby enhancing the study’s clinical translational value. Another strength of this study is the robustness of its results. Despite some statistical heterogeneity, sensitivity analysis indicated that the core pooled effect size remained stable and statistically significant after excluding individual studies, indicating that no single study drove the overall estimate, though the high heterogeneity and very low certainty of evidence warrant cautious interpretation.

Despite these strengths, this study has some unavoidable limitations. First, the number of included original studies is relatively small (11 articles, 11 datasets), and some studies have small sample sizes, which may have impacted the statistical power of the meta-analysis and reduced the generalizability of the conclusions. Second, while we explored sources of heterogeneity through subgroup analysis, the included studies still exhibited variation in intervention protocols, such as diverse soft Chinese martial arts styles (e.g., Baduanjin, Tai Chi), inconsistent frequencies and intensities, and a lack of uniform standards for instructors. These factors make it difficult to precisely attribute effects to a specific martial arts style or intervention element.

In addition, baseline depressive scores showed some between-group differences in a few included studies (see **Table 1**); although formal baseline testing was not consistently reported in the primary studies, these imbalances could not be excluded and should be considered when interpreting the pooled estimates.

Third, meta-regression was considered to explore potential sources of heterogeneity (e.g., intervention duration, frequency, session length). However, because only 11 studies were included, a meta-regression would have insufficient statistical power and a high risk of spurious findings. Therefore, we relied on subgroup analyses and leave-one-out sensitivity analyses to examine heterogeneity. Although these methods did not fully explain the high I^2^ (82.6%), they represent the most appropriate analytical approach given the limited number of studies.

Fourth, the original studies included in this meta-analysis primarily relied on self-report scales to assess depressive mood, which may introduce self-report bias. The lack of objective physiological or neurobiological indicators (e.g., cortisol, EEG, neuroimaging) to verify mental health changes limits a deeper understanding of the potential mechanisms. Finally, this meta-analysis primarily focused on the short-term effects of the intervention and lacked an assessment of its long-term impact on the mental health of college students, which awaits future investigation. Moreover, with regard to data completeness, 10 of the 11 included studies provided complete data on post-intervention means and SDs, and SDs for the remaining study (30) were imputed from the reported 95% confidence intervals using a standard formula; no studies were excluded due to missing SDs. A sensitivity analysis excluding this single study was not performed, because the proportion of imputed data was minimal (1/11, ~9.1%) and the imputation was based on directly reported CIs; the use of imputed data may therefore introduce additional, albeit limited, uncertainty into the pooled estimate, although the direction and magnitude of any such bias are difficult to predict.

Furthermore, the included studies did not consistently report on contextual and pedagogical factors such as instructor qualifications, training environment, interpersonal climate, or safeguarding procedures. These factors may substantially influence the mental health outcomes of martial arts practice and represent an important avenue for future research. Our findings should not be interpreted as implying that martial arts practice is inherently beneficial; rather, the effects likely vary according to how, where, by whom, and with whom the practice is delivered (13, 20, 21).

Fifth, limitations related to the search strategy. Although we systematically searched seven major electronic databases and manually screened reference lists, we did not search grey literature sources (e.g., conference abstracts, preprints, dissertations not indexed in our databases) or trial registries (e.g., ClinicalTrials.gov, WHO ICTRP). This was a protocol-driven decision, but it may have introduced publication bias and missed unpublished or ongoing trials. Forward citation tracking was also not performed. These limitations are acknowledged, and future reviews should consider broader searches to enhance completeness. Beyond statistical significance, the clinical meaningfulness of the observed effect deserves consideration. Although minimal clinically important differences (MCIDs) have not been firmly established for the depression scales used in this meta-analysis (SDS, BDI, SCL-90) specifically in college student populations, the magnitude of the pooled effect (SMD = –0.71) is generally considered moderate-to-large in psychosocial interventions. Moreover, several individual studies reported mean differences exceeding 10 points on the SDS or BDI, which likely exceeds the threshold that students would perceive as a meaningful improvement in their depressive symptoms. Therefore, the beneficial effect of martial arts on depressive symptoms appears not only statistically significant but also likely clinically relevant. Future research should establish population-specific MCID values to enable more precise quantification of clinically meaningful change.

The inclusion of the three master's theses retrieved from CNKI (30, 33, 34) also warrants consideration. Unlike peer-reviewed journal articles, these dissertations were not subjected to anonymous double-blind peer review, and their methodological and reporting quality may therefore be more variable. Two considerations mitigate this concern: (1) a sensitivity analysis excluding these three studies yielded a pooled effect that remained statistically significant (SMD = –0.68, 95% CI: –1.25 to –0.11, p = 0.019) and consistent with the overall estimate (SMD = –0.71), indicating that our findings do not depend on their inclusion; and (2) all included studies, including the dissertations, were appraised using the same Cochrane Risk of Bias tool, thereby ensuring a uniform quality assessment across study types. Taken together, the inclusion of dissertations is unlikely to have materially affected our core conclusions.

### 4.3 Directions for future research

This meta-analysis has provided preliminary evidence for the efficacy of soft Chinese martial arts interventions in alleviating depressive mood among college students, yet it has also highlighted existing research limitations. Based on these findings, we propose the following key directions for future research:

First, Improve Research Design: Conduct larger, multi-center RCTs with robust active control groups to enhance statistical power and generalizability.

Second, Refine the Understanding of Potential Moderating Effects: Systematically design intervention protocols with varying “doses” (intensity, total hours) to precisely determine the optimal duration, frequency, and session length for maximal therapeutic benefit.

Third, Explore Differential Effects: Compare the effects of different martial arts styles (e.g., Tai Chi vs. hard martial arts) on depressive mood to identify which forms are most suitable for mental health interventions.

Fourth, Investigate Underlying Mechanisms: Use a multidisciplinary approach with objective indicators, such as neuroimaging (fMRI, fNIRS), to provide a deeper understanding of the biological and neural pathways through which martial arts exert their antidepressant effects.

Fifth, Assess Long-Term Effects: Implement long-term follow-up studies to evaluate the sustained effects of soft Chinese martial arts interventions and explore strategies for long-term mental health management.

Sixth, Diversify Study Populations: Extend research to include college students from diverse cultural backgrounds and socioeconomic statuses to enhance the generalizability of the findings.

Seventh, Report Contextual and Pedagogical Factors: Future RCTs should systematically report contextual and pedagogical variables—such as instructor qualifications, safeguarding procedures, training environment and interpersonal climate, partner selection, task progression, and regulation of training intensity—so that the conditions under which soft Chinese martial arts interventions confer mental health benefits can be identified (13, 20, 21).

### 4.4 GRADE certainty of evidence

Using the GRADE framework, the certainty of evidence for the primary outcome (depressive symptoms) was rated as very low because of: (1) risk of bias (unclear allocation concealment and lack of blinding in most studies), (2) inconsistency (substantial heterogeneity, *I^2^* = 82.6%, not fully explained by subgroup analyses), and (3) possible publication bias (Egger’s test *p* = 0.397, low power due to small number of studies). No upgrading factors (large effect, dose–response) were applied because the pooled SMD of –0.71 was derived from small studies with high heterogeneity and the observed subgroup differences were exploratory. Thus, the overall certainty of evidence is very low, meaning that the true effect is likely to be substantially different from the estimate, and further research is very likely to change our confidence in the estimate.

## 5 Conclusion

This meta-analysis provides preliminary evidence that soft Chinese martial arts interventions, specifically Tai Chi and Baduanjin, may reduce depressive symptoms in college students. The observed effect (SMD = –0.71) corresponds to a moderate-to-large effect size by conventional benchmarks; however, this should be interpreted with caution given the high heterogeneity and very low certainty of evidence. Using the GRADE framework, the certainty of evidence was rated as very low. The findings offer initial support for using soft Chinese martial arts as a non-pharmacological intervention, but further rigorous RCTs are needed to confirm the effect, explore moderators, and assess long-term outcomes.

## Data Availability

The original contributions presented in the study are included in the article/**Supplementary Material**. Further inquiries can be directed to the corresponding author.
