# Supplementary material for: Effect of soft Chinese martial arts on depressive symptoms among college students: a systematic review and meta-analysis
Source: Front Psychiatry. 2026 Oct 20;17:1850402. doi: 10.3389/fpsyt.2026.1850402 (PMC13729382; doi:10.3389/fpsyt.2026.1850402)
Supplement: Supplementary file 1 [file Table1.docx]

**Supplementary Table S1. Complete database-specific search strategies for the seven databases**

*All searches were conducted on July 31, 2025, with no lower date restriction, and were restricted to English- and Chinese-language publications. Boolean operators (AND, OR) were used. Field tags, date limits, and language limits are shown for each database as executed.*

| **Database** | **Search strategy (syntax, field tags, date limits, language limits)** |
| --- | --- |
| **PubMed/MEDLINE** | ("martial arts"[MeSH Terms] OR "martial arts"[Title/Abstract] OR "kung fu"[Title/Abstract] OR "wushu"[Title/Abstract] OR "tai chi"[Title/Abstract] OR "taiji"[Title/Abstract] OR "taijiquan"[Title/Abstract] OR "qigong"[Title/Abstract] OR "baduanjin"[Title/Abstract]) AND ("students"[MeSH Terms] OR "college students"[Title/Abstract] OR "university students"[Title/Abstract] OR "undergraduate"[Title/Abstract] OR "postgraduate"[Title/Abstract] OR "higher education"[Title/Abstract]) AND ("depression"[MeSH Terms] OR "depressive symptoms"[Title/Abstract] OR "depressed mood"[Title/Abstract] OR "mood disorders"[MeSH Terms] OR "mental health"[Title/Abstract] OR "psychological distress"[Title/Abstract]) AND ("0001/01/01"[Date - Publication] : "2025/07/31"[Date - Publication]) |
| **Embase** | ('martial arts'/exp OR 'martial arts':ti,ab OR 'kung fu':ti,ab OR 'wushu':ti,ab OR 'tai chi':ti,ab OR 'taijiquan':ti,ab OR 'qigong':ti,ab OR 'baduanjin':ti,ab) AND ('student'/exp OR 'college student':ti,ab OR 'university student':ti,ab OR 'undergraduate':ti,ab OR 'postgraduate':ti,ab) AND ('depression'/exp OR 'depressive symptom':ti,ab OR 'depressed mood':ti,ab OR 'mood disorder':ti,ab OR 'mental health':ti,ab OR 'psychological distress':ti,ab) AND ([english]/lim OR [chinese]/lim) AND [2025-07-31]/sd |
| **Web of Science (Core Collection)** | TS=("martial arts" OR "kung fu" OR "wushu" OR "tai chi" OR "taiji" OR "taijiquan" OR "qigong" OR "baduanjin") AND TS=("college students" OR "university students" OR "undergraduate*" OR "postgraduate*" OR "higher education") AND TS=("depression" OR "depressive symptom*" OR "depressed mood" OR "mood disorder*" OR "mental health" OR "psychological distress"). Timespan: all years to 2025-07-31. Language: English or Chinese. |
| **Scopus** | TITLE-ABS-KEY("martial arts" OR "kung fu" OR "wushu" OR "tai chi" OR "taijiquan" OR "qigong" OR "baduanjin") AND TITLE-ABS-KEY("college students" OR "university students" OR "undergraduate" OR "postgraduate") AND TITLE-ABS-KEY("depression" OR "depressive symptoms" OR "depressed mood" OR "mood disorder" OR "mental health" OR "psychological distress") AND PUBYEAR < 2026 AND (LANGUAGE(English) OR LANGUAGE(Chinese)) |
| **CENTRAL (Cochrane Library)** | #1 MeSH descriptor: [Martial Arts] explode all trees  #2 ("martial arts" OR "kung fu" OR "wushu" OR "tai chi" OR "taijiquan" OR "qigong" OR "baduanjin"):ti,ab,kw  #3 MeSH descriptor: [Students] explode all trees  #4 ("college students" OR "university students" OR "undergraduate" OR "postgraduate"):ti,ab,kw  #5 MeSH descriptor: [Depression] explode all trees  #6 ("depression" OR "depressive symptoms" OR "depressed mood" OR "mood disorder" OR "mental health" OR "psychological distress"):ti,ab,kw  #7 (#1 OR #2) AND (#3 OR #4) AND (#5 OR #6). Date limit: inception to July 2025. |
| **PsycINFO** | S1 DE "Martial Arts" OR TI/AB ("martial arts" OR "kung fu" OR "wushu" OR "tai chi" OR "taijiquan" OR "qigong" OR "baduanjin")  S2 DE "College Students" OR TI/AB ("college students" OR "university students" OR "undergraduate" OR "postgraduate")  S3 DE "Major Depression" OR TI/AB ("depression" OR "depressive symptoms" OR "depressed mood" OR "mood disorder" OR "mental health" OR "psychological distress")  S4 S1 AND S2 AND S3. Date limit: 1967 to July 2025. Language: English or Chinese. |
| **CNKI (China National Knowledge Infrastructure)** | SU=("武术" OR "太极拳" OR "太极" OR "气功" OR "八段锦" OR "功夫") AND SU=("大学生" OR "高校学生" OR "在校大学生" OR "大学生群体") AND SU=("抑郁" OR "抑郁症" OR "抑郁情绪" OR "抑郁症状" OR "心理健康" OR "心理困扰"). Date limit: inception to July 31, 2025. Language: Chinese. |

*Note: MeSH = Medical Subject Headings; TS = Topic field (Web of Science); TITLE-ABS-KEY = title, abstract and keywords fields (Scopus); ti,ab = title and abstract fields; ti,ab,kw = title, abstract and keywords fields (Cochrane); DE = descriptor/thesaurus term (PsycINFO); SU = subject field (CNKI).*
